# The Multifaceted Role of Signal Peptide-CUB-EGF Domain-Containing Protein (SCUBE) in Cancer

**DOI:** 10.3390/ijms231810577

**Published:** 2022-09-12

**Authors:** Shashank Kumar, Kumari Sunita Prajapati, Sanjay Gupta

**Affiliations:** 1 Molecular Signaling & Drug Discovery Laboratory, Department of Biochemistry, Central University of Punjab, Bathinda 151401, India; 2 Department of Urology, Case Western Reserve University, Cleveland, OH 44106, USA; 3 The Urology Institute, University Hospitals Cleveland Medical Center, Cleveland, OH 44106, USA; 4 Department of Pathology, Case Western Reserve University, Cleveland, OH 44106, USA; 5 Department of Pharmacology, Case Western Reserve University, Cleveland, OH 44106, USA; 6 Department of Nutrition, Case Western Reserve University, Cleveland, OH 44106, USA; 7 Division of General Medical Sciences, Case Comprehensive Cancer Center, Cleveland, OH 44106, USA; 8 Department of Urology, Louis Stokes Cleveland Veterans Affairs Medical Center, Cleveland, OH 44106, USA

**Keywords:** SCUBE, angiogenesis, signaling, tumor suppressor, oncogene, metastasis, biomarkers

## Abstract

Signal peptide, CUB, and EGF-like domain-containing proteins (SCUBE) are secretory cell surface glycoproteins that play key roles in the developmental process. SCUBE proteins participate in the progression of several diseases, including cancer, and are recognized for their oncogenic and tumor suppressor functions depending on the cellular context. SCUBE proteins promote cancer cell proliferation, angiogenesis, invasion, or metastasis, stemness or self-renewal, and drug resistance. The association of SCUBE with other proteins alters the expression of signaling pathways, including Hedgehog, Notch, TGF-β/Smad2/3, and β-catenin. Further, SCUBE proteins function as potential prognostic and diagnostic biomarkers for breast cancer, renal cell carcinoma, endometrial carcinoma, and nasopharyngeal carcinoma. This review presents key features of SCUBE family members, and their structure and functions, and highlights their contribution in the development and progression of cancer. A comprehensive understanding of the role of SCUBE family members offers novel strategies for cancer therapy.

## 1. Introduction

**Figure 1:**
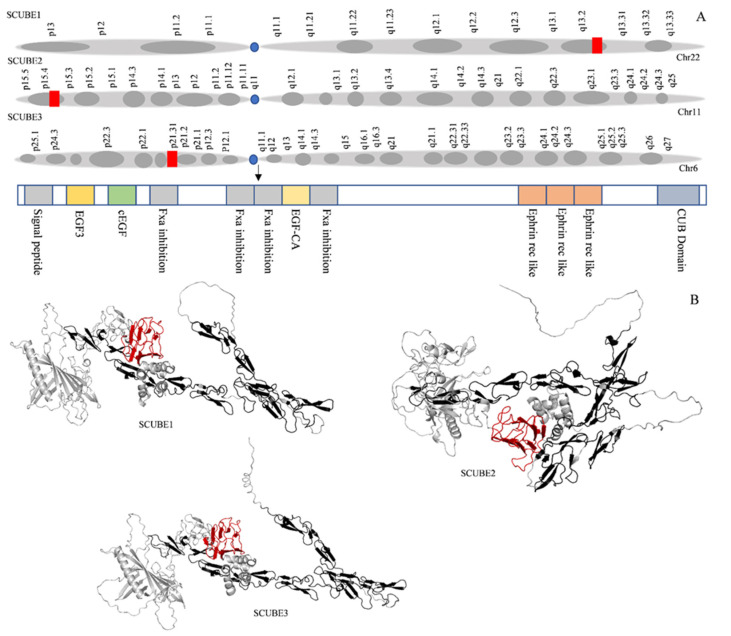
(**A**) Location of SCUBE1, SCUBE2, and SCUBE3 on their respective chromosomes and common arrangement of structural domains in SCUBE proteins. SCUBE family proteins as surface glycoprotein. They are composed of an N-terminal region, signal peptide sequence (SPS), nine EGF-like repeats (E), a spacer region (SR), three cysteine rich repeats (CR), a CUB domain, and a C-terminal region. (**B**) Structural discrepancy of SCUBE family protein. Grey represents whole protein structure, black EGF-like repeats-Ca binding site, and red is the CUB domain.

**Figure 2:**
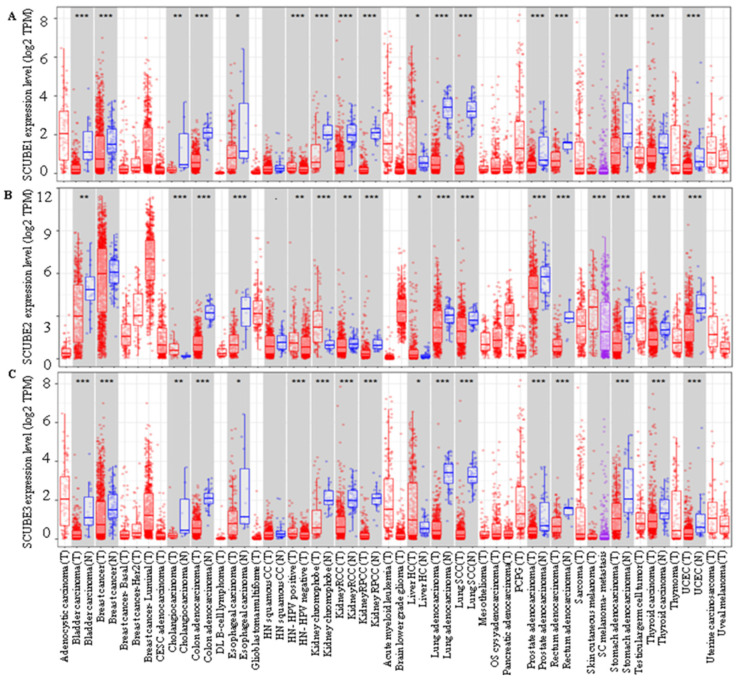
Expression of SCUBE family members in various human tumors. (**A**) SCUBE1, (**B**) SCUBE2, and (**C**) SCUBE3 expression in different cancer types in comparison to adjacent normal tissue. Data extracted from TCGA database determined by tumor immune estimation resource (TIMER) presented as log2-TPM. TPM denotes transcript count per million.

Signal peptide-CUB (complement C1r/C1s, Uegf, and Bmp1)-EGF (epidermal growth factor)-domain-containing proteins (SCUBE) are evolutionarily conserved, secreted and cell surface glycoproteins that comprise three family members, SCUBE1, SCUBE2, and SCUBE3 [1]. These members were identified and termed in the order of their discovery in mammals. SCUBE family contains an organized protein structure with recognized structural motifs consisting of an N-terminal signal peptide sequence, followed by nine copies of EGF-like repeats of the calcium-binding class, a spacer region, three cysteine-rich motifs, and one CUB domain at the C-terminus. The EGF-like repeats and CUB domain are linked by a spacer region that participates in ligand binding and protein–protein interactions. The SCUBE family plays a critical role in the normal tissue development of the nervous system, limb buds, surface ectoderm, somites, and gonads [2,3]. SCUBE1 is present on chromosome 22q13 and is composed of 988 amino acids with a molecular weight of 107.91 kDa. It is a secretory cell-associated protein localized into human vascular endothelium and a novel adhesive molecule in human platelets [4]. SCUBE1 is stored in platelet α-granules where, during post activation, it is transferred to the cell surface and released via proteolysis. SCUBE1 is commonly associated with pathological conditions such as thrombus, atherosclerotic plaque, inflammation, and hypoxia-related disorders [4,5]. SCUBE2 consists of 999 amino acids with a molecular weight of 109.96 kDa present on chromosome 11. SCUBE2 protein was first reported in zebrafish as an essential mediator of Hedgehog (Hh) signaling. Later, it was reported to regulate Hh signaling in humans. Jakob et al. (2014) reported that SCUBE2 is required for the release of sonic hedgehog (Shh) from the cell membrane. This function of SCUBE2 is mediated by the CUB domain participating as a regulator of proteolytic activity [6]. Furthermore, SCUBE2 functions as a coreceptor of vascular endothelial growth factor receptor 2 (VEGFR2) resulting in increased VEGF signaling and consequently promoting angiogenesis [7]. Another SCUBE family protein, SCUBE3, was first identified in the umbilical vein and in human endothelial cells [8]. It plays a critical role in cell differentiation, recognition, and modeling. SCUBE3 is present on chromosome 6 with 993 amino acids with a molecular weight of 109.28 kDa. The chromosomal location of SCUBE1, SCUBE2, and SCUBE3, and the arrangement of their structural domains and structural divergence are shown in Figure 1. SCUBE family proteins participate in the development of various human diseases, including cancer (Table 1), and are differentially expressed in tumors compared to normal tissue (Figure 2).

## 2. Defects in SCUBE Family in Cancer

**Figure 3:**
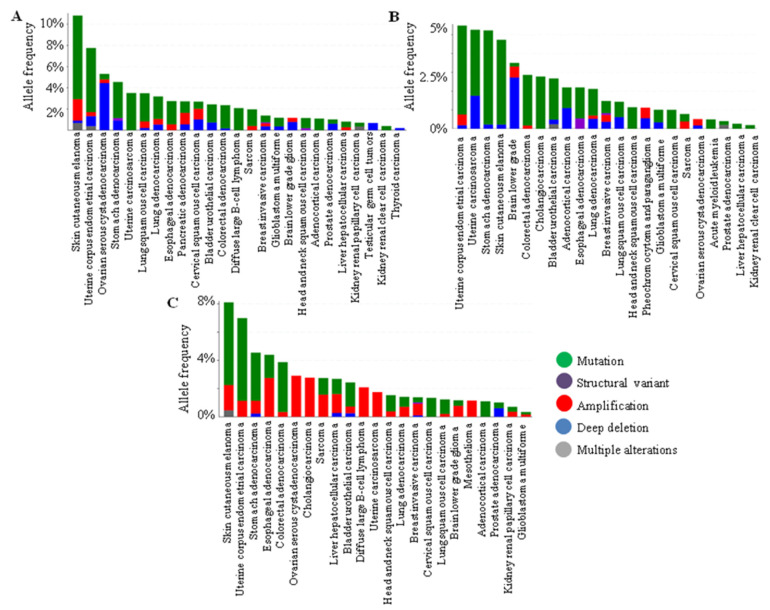
Genomic alteration in SCUBE family genes in various human cancers. Mutation and CNA data for SCUBE analyzed from 32 studies and 10,967 patient’s samples using cBioportal. (**A**) Data show the highest SCUBE1 gene alteration in skin cutaneous melanoma; (**B**) SCUBE2 shows the highest gene alterations in uterine corpus endometrial carcinoma; (**C**) SCUBE3 exhibits overexpression in skin cutaneous melanoma. Gene alteration is reported as amplification, deep deletion, mutation, structural variation, or multiple alterations.

Cancer genomics data (mutation and copy number alterations; CNA) for the SCUBE family, analyzed from 32 studies comprising 10,967 patient’s samples and downloaded through cBioPortal, demonstrated the highest SCUBE1 gene alteration in skin cutaneous melanoma and carcinomas of the uterine corpus, endometrium, ovary, stomach, lungs, breast, colon, and kidneys. SCUBE2 exhibited the highest gene alterations in the uterine corpus endometrial carcinoma and uterine carcinosarcoma, followed by carcinomas of the stomach, skin, brain, bladder, lungs, breasts, ovaries, prostate, and pancreas [9,18,19,20,21,22,23,24,25,26,27]. SCUBE3 showed increased alterations consisting of mutation and amplification in skin cutaneous melanoma, followed by uterine corpus endometrial carcinoma, malignancies of the stomach, esophagus, colon–rectum, ovaries, liver, bladder, breasts, lungs, cervix, brain, prostate, and glioblastoma (Figure 3).

## 3. SCUBE Association with Other Proteins

**Figure 4:**
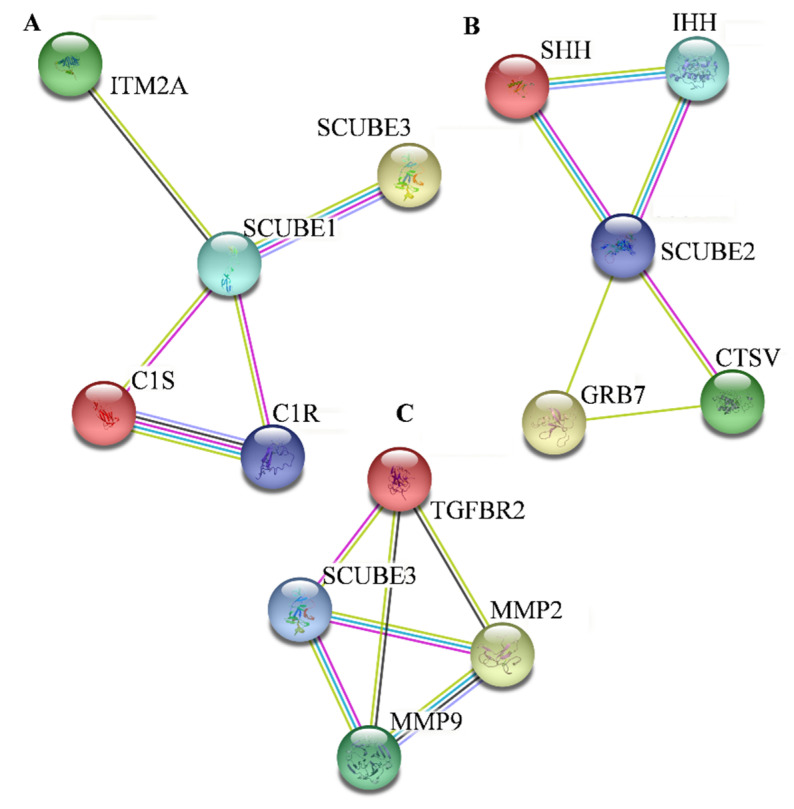
Protein–protein interaction network with SCUBE family and other proteins on STRING database. These interactions with other proteins were experimentally studied. (**A**) SCUBE1; (**B**) SCUBE2; (**C**) SCUBE3.

SCUBE family associations with other proteins were derived from STRING database. Network analysis showed the interaction of 10 proteins with SCUBE1, SCUBE2, and SCUBE3 (Figure 4). A direct interaction of SCUBE1 with SCUBE3 and with other proteins includes C1S, C1R, and ITM2A. SCUBE2 directly interacts with CTSV, GRB7, IHH, and SHH proteins. Furthermore, SCUBE3 exhibited direct interaction with MMP2, MMP9, and TGFBR2 proteins. The interaction and association of SCUBE family with other proteins is shown in Table 2, which is based on curated databases, experimental determination, gene neighborhood, and text mining.

## 4. Emerging Role of SCUBE Family in Human Cancers

The aberrant expression of the SCUBE family plays a key role in cancer-cell proliferation, apoptosis, invasion, and migration in both in vitro and in vivo studies. SCUBE family proteins interact with other proteins and thus regulate their expression. SCUBE members positively modulate bone morphogenetic protein (BMP) signaling via inducing the specific interaction between BMP and BMP type I receptors affecting bone growth. Functionally, the SCUBE family maintains cellular signaling and regulates the expression of target genes via transcriptional or post-transcriptional mechanisms. The altered expression of SCUBE in cancer is linked to clinical outcome, highlighting the significant role that they may play as novel diagnostic and prognostic biomarkers, and as targets for cancer therapeutics.

### 4.1. Role of SCUBE as a Tumor Suppressor

SCUBE1 is associated with the proteins involved in angiogenesis, including platelet-derived growth factor D (PDGF-D), transforming growth factor-beta (TGF-β), and hedgehog (Hh). The mutations and mis-regulation of Hh signaling pathway components (PTCH1 and SMO) are associated with various cancer types [9]. On the other hand, SCUBE2 expression is regulated and coordinated by two distinct mechanisms, namely, the BMP and β-catenin pathways [18]. Antagonizing BMP and suppressing the β-catenin pathway exerts an inhibitory effect on breast-cancer cells. This phenomenon occurs on the C-terminal of the CUB domain that directly binds to the BMP and restricts its activity in an autocrine manner. In parallel, the N-terminal EGF-like repeats mediate cell-to-cell hemophilic adhesions in a calcium-dependent manner with further interaction with E-cadherin [18]. Furthermore, SCUBE2 functions as a tumor suppressor in nonsmall-cell lung cancer (NSCLC), glioma, and breast and colorectal cancer through the inhibition of the cell proliferation, migration, and invasion of tumor cells [18,22,23,24,36]. Using bioinformatics analysis, a study demonstrated that the co-expression of SCUBE2 with N-acetyltransferase (NAT1) results in the alteration of several genes that play a critical role in triple-negative breast-cancer (TNBC) progression [37,38]. Another study identified the association of chromosome 19 miRNAs and SCUBE2 in the progression of TNBC subtype [39]. Cheng et al. (2009) reported that altered SCUBE2 plays a significant role in breast-cancer cell proliferation and progression. This study revealed that the overexpression of the COOH terminal of SCUBE2 reduces MCF-7 breast-cancer cell growth. The overexpression of SCUBE2 protein functions as a BMP antagonist that suppresses cell proliferation in vitro and decreases tumor growth in an MCF-7 xenograft nude mouse model [36]. Genome wide approaches such as methylated DNA immunoprecipitation (MEDIP) and whole-genome array analysis, in combination with high-density expression analysis, identified SCUBE3 as a frequently methylated and silenced gene in renal cell carcinoma (RCC), and thus a candidate tumor suppressor gene. A study reported that SCUBE3 tumor methylation was significantly associated with increased risk of cancer relapse and cancer-related death. The RNAi knockdown of SCUBE3 was associated with an advantage in anchorage-independent growth [27].

### 4.2. SCUBE in Cell Proliferation and Cancer Progression

Accumulating evidence demonstrated that alteration in SCUBE expression promotes tumor growth in a cellular-context-dependent manner [7,9,19,22,23,24,25,26,27]. Liang et al. (2015) reported the upregulation of SCUBE3 at both the transcript and the protein level in osteosarcoma cells (OS) and tissues compared with normal osteoblasts. The knockdown of SCUBE3 by siRNA significantly inhibited the proliferation of OS [26]. A study reported the functional role of SCUBE3 in regulating the calcium ion pathway and their interactive association in human carcinogenesis through integrated network analysis. The group reported that lncRNA (CTB-113D17.1) competitively binds to hsa-miR-590-5p and facilitates SCUBE3 expression in OS [40,41]. Han et al. (2018) reported the overexpression of SCUBE3 in salivary adenoid cystic carcinoma (SACC) patients in comparison to a normal noncancerous gland. Dual-luciferase assay showed SCUBE3 to be a direct target of hsa-miR-885-5p [25]. A recent study demonstrated an association between increased SCUBE3 expression and low E-cadherin levels in high-histological-grade breast cancer, conforming a significant role of SCUBE3 in cancer progression and poor prognosis. The group also found an association of SCUBE3 expression with stage-specific breast tumors and subtypes. SCUBE3 expression was higher in TNBC in comparison to luminal, HER2+, and other breast-cancer subtypes [42]. Another study reported that the forced expression of SCUBE1 reduces pro-tumorigenic activity in prostate-cancer-associated fibroblasts (CAFs) in a pre-clinical model [43].

### 4.3. SCUBE in Regulating Angiogenesis

SCUBE proteins, primarily SCUBE2 upregulation, participate in the regulation of tumor angiogenesis. The process of the formation of new blood vessels is referred to as angiogenesis, which is critical for tumor growth and metastasis. In this context, VEGF interacts with VEGFR2 and activates endothelial cells that induce a cascade of expression of downstream molecules such as phospholipase C, PI3K/Akt, and γ-MAPK. These signaling cascades together promote endothelial cell proliferation, migration, and vessel formation [7]. Lin et al. (2018) demonstrated the coreceptor performing activity of SCUBE2 with VEGFR2. Upon the induction of hypoxia, SCUBE2 promotes VEGF binding to its receptor to increase angiogenesis. EC-specific Scube2-knockout (EC-KO) and SP.B1 (anti-SCUBE2 monoclonal antibody) inhibited vascular tumor density and xenograft tumor growth, increased apoptosis, and reduced tumor-cell proliferation in a mouse model. The mechanism behind reducing tumor angiogenesis is the binding of SP.B1 to SCUBE2, which internalizes it to lysosomal degradation by preventing binding to VEGF and the activation of downstream signaling of VEGF. The binding of SP.B1 to SCUBE2 inhibited VEGFR2 phosphorylation and Akt/MAPK activation [44]. This study was designed to develop anti-SCUBE2 therapy against tumor angiogenesis.

### 4.4. SCUBE in Invasion and Metastasis

Jiang et al. (2019) reported the relationship between SCUBE1 and miR-22 overexpression in a transcriptomic study. miR-22 acts as a tumor suppressor and has inhibitory effects on tumor growth, migration, and invasion. The group constructed miR-22-overexpressing cells found that SCUBE1 upregulation in the constructed cells is not the direct target of miR-22 and exhibited significantly increased apoptosis. Moreover, the study showed WRNIP1 to be a direct target of miR-22. The overexpression of miR-22 increases the radiosensitivity of NSCLC cells by targeting WRNIP1 [45].

Yang et al. (2018) demonstrated the significant downregulation of SCUBE2 protein in NSCLC cell lines and human tissues. The group demonstrated that the forced expression of SCUBE2 markedly inhibited cell proliferation and induced apoptosis in NSCLC cells, whereas the upregulation of SCUBE2 altered NSCLC cell migration and invasion by inhibiting the sonic hedgehog signaling pathway. The forced expression of SCUBE2 altered the expression of the Smo, Shh, Gli1, and ptch1 proteins [22]. Guo et al. (2017) reported low expression of SCUBE2 in glioma cell lines and tissues. The overexpression of SCUBE2 through transfection with pcDNA3.1-SCUBE2 into glioma U87 and A172 cells inhibited proliferation, migration, and invasion through the inhibition of the activity of the Shh signaling pathway. SCUBE2 overexpression substantially inhibited Gli1 expression in glioma cells. SCUBE2 plays a significant role in breast-cancer regulation [24]. The expression of SCUBE2 and other breast-cancer-associated genes was clinically utilized to determine a predictive score in adjuvant guided treatment for breast-cancer patients [15,46,47]. SCUBE2 expression regulates epithelial-to-mesenchymal (EMT) transition in breast-cancer cells. The ectopic expression of SCUBE2 reverses EMT transition and inhibits aggressiveness in breast-cancer cells (MDA-MB-231) by forming epithelial E-cadherin-containing adherens junctions. The -cadherin-containing adherens junction is regulated by the β-catenin-SOX-mediated induction of FOXA1 (a positive regulator of E-cadherin). This mechanism decreases E-cadherin-mediated EMT transition in tumor cells. The direct expression of SCUBE2 is sufficient to inhibit TGF-β-mediated EMT induction in breast-cancer cells [48]. Shen et al. (2020) identified the relationship among circular RNA circ_SETD2 (circ_SETD2), SCUBE2, and miR-155-5p in breast cancer. Both circ_SETD2 and SCUBE2 were downregulated, whereas miR-155-5p was upregulated in breast cancer. The overexpression of circ_SETD2 and SCUBE2 increases cell-cycle arrest, induces apoptosis, and inhibits cell proliferation, migration, and invasion in breast-cancer cells. Circ_SETD2 binds competitively to miR-155-5p and upregulates SCUBE2 expression, thereby inhibiting breast-cancer progression [49]. SCUBE2 is downregulated in colorectal cancer. The low expression of SCUBE2 is associated with advanced clinical stage, higher-histological-grade tumor, lymph nodes, and distant metastasis in colorectal-cancer (CRC) patients. The restoration of SCUBE2 reduced cell proliferation and colony formation, migration, and invasion in an in vitro model [23]. The molecular mechanism behind SCUBE2 regulating CRC cell migration and invasion needs to be further evaluated. Wang et al. (2018) reported a lower expression of SCUBE2 at both the mRNA and protein levels in gastric cancer. The decreased expression of SCUBE2 is associated with lymph-node metastasis, high-grade tumors, advanced clinical stage, higher histological grade, distant-site metastasis, and vascular invasion [50]. Parris et al. (2014) predicted the association of SCUBE2 with CBX2 and STK32B in the appearance of clinical–pathological characteristics, including increased tumor size, metastatic spread to cervical lymph nodes, and peritumoral inflammatory infiltration. Islam et al. (2016) identified that Shh signaling has the potential to increase tumorigenicity and stemness via EMT activation in bladder cancer through the switching and functional activation of CD133, Sox2, Nanog, and Oct4 [51]. It would be interesting to know whether the SCUBE family plays a significant role in bladder-cancer progression. Furthermore, SCUBE2 functions as a tumor suppressor gene and is hypermethylated in breast tumors. Treatment with (-)-epigallocatechin-3-gallate (EGCG), a major polyphenolic constituent of green tea, significantly reversed SCUBE2 methylation by decreasing the expression of DNA methyltransferase (DNMT). An EGCG-mediated increase in SCUBE2 expression inhibits vimentin expression, resulting in the suppression of breast-cancer cell migration and invasion [52].

The upregulation of SCUBE3 was observed in NSCLC compared to adjacent normal tissues. SCUBE3 regulates the expression of several oncogenes in an autocrine or paracrine manner. SCUBE3 regulates the transcriptional and translational expression of the TGF-β/Smad2/3 signaling pathway responsible for the increased invasion of lung-cancer cells. MMP2 and MMP9 cleave SCUBE3 into two fragments *viz*. N-terminal EGF-like repeats and the C-terminal CUB domain. C-terminal fragments having CUB domain either by themselves or together with TGF-β1 bind to the TGF-βII receptors and activate the TGF-β receptor/Smad2/3 signaling pathway. The activation of Smad2/3 through phosphorylation leads to the expression of downstream target genes. These include TGF-βI, slug and snail, MMP-2, MMP-9, PAI-1, and VEGF, which further promote epithelial-to-mesenchymal transition, invasion, metastasis, extracellular-matrix deposition, and angiogenesis in lung-cancer cells. SCUBE3 knockdown expression reduced the metastatic potential of NSCLC and tumor growth in an in vivo model [35]. Microarray analysis revealed that SCUBE3 knockdown lowers the expression of genes controlling EMT and early angiogenesis in lung carcinoma [53]. Zhao et al. (2013) investigated the clinical–pathological and prognostic significance of SCUBE3 in NSCLC tumors. Furthermore, the higher expression of SCUBE3 was positively correlated with advanced-stage tumors and lymph-node metastasis. The overexpression of SCUBE3 was also associated with the reduced expression of E-cadherin, and a higher expression of vimentin in NSCLC tumors [54].

### 4.5. SCUBE in the Maintenance of Cancer Stemness and Drug Resistance

**Figure 5:**
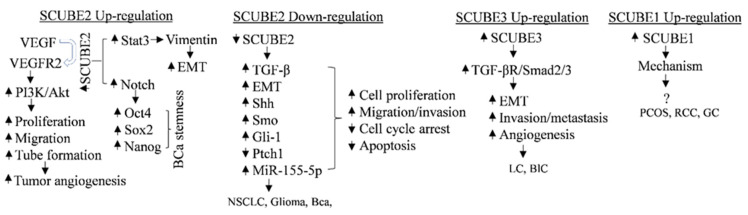
Mechanism of SCUBE family in cancers and cancer stem cells in a contextual manner. VEGF, vascular endothelial growth factor; VEGFR2, vascular endothelial growth factor receptor 2; PI3K/Akt, phosphoinositide 3-kinase/protein kinase B; EMT, epithelial–mesenchymal transition; BCa, breast-cancer; Shh, sonic hedgehog; Ptch, patched homolog; Smo, smoothened; Gli, Gli family zinc finger; MMP-2/9, matrix metalloproteinase 2/9; Stat3, signal transducer and activator of transcription factor 3; TGF, transforming growth factor-β; NSCLC, nonsmall-cell lung cancer; LC, lung cancer; BlC, bladder cancer; PCOS, polycystic ovary syndrome; RCC, renal-cell cancer; GC, gastric cancer.

SCUBE2 functions as a novel breast tumor suppressor [18] and possesses an oncogenic role in breast-cancer stem cells [19]. The overexpression of SCUBE2 in breast-cancer stem cells increases TNBC aggressiveness via modulating the Stat3 and Notch signaling pathway. SCUBE2 upregulation increases the anchorage-independent cell growth, proliferation, migration, and invasion of tumor cells. SCUBE2 overexpression also increases cancer stem cell differentiation and self-renewal potential via an increase in the expression of ALDH1, Oct4, Sox2, and Nanog, and mammosphere formation, and reduces sensitivity to paclitaxel chemotherapy [19]. Kallarakal et al. (2020) proposed SCUBE2, ELF5, and NFIB as a 3-signature gene panel biomarker to predict taxane-based neoadjuvant response in breast cancer [55]. Recently, SCUBE2 was identified as a drug resistance gene that could be a promising tool for risk classification in ER-positive and HER2-negative breast-cancer patients [56]. The expression of SCUBE family members in cancers and cancer stem cells, and their associated signaling pathways and targets are shown in Table 3; the mechanism is shown in Figure 5.

### 4.6. SCUBE and Immune Response

Erol et al. (2018) investigated the role of circulating SCUBE1 in lean, young, and glucose-tolerant women having polycystic ovary syndrome (PCOS), and healthy individuals. No significant association was noted between higher SCUBE 1 level and PCOS parameters. However, an increase in SCUBE1 was under the influence of proinflammatory cytokines such as tumor necrosis factor-α (TNF-α) and interleukin-1β (IL-1β) [57,60]. Another study demonstrated that the lower expression of SCUBE1 in multiple myeloma patients could be responsible for an increased risk for arterial thrombosis, although the association remains unestablished. The study concluded that lower expression of SCUBE1 may be possible due to defective platelets or increased TNF-α expression in multiple myeloma patients [61].

### 4.7. SCUBE as a Biomarker

Cancer patients show an increased risk of developing thrombosis, and a continuous activation of the coagulation process. Some tumors have coagulation dysfunction that generates procoagulant substances [62]. D-dimer is a well-known biomarker responsible for coagulation activation and fibrinolysis. D-dimer is independently associated with increased risk of thrombosis, which remains a major factor for breast-cancer-related deaths in women. SCUBE1 has been investigated in veins, arteries, and microvessels, and its higher expression was observed in the plasma of acute coronary and ischemic syndrome [63]. Topcu et al. (2015) investigated the expression of SCUBE1 in breast-cancer patients and it compared with that of healthy individuals. SCUBE1 expression was significantly higher in the serum of HER2-positive breast-cancer patients than that in the HER2-negative phenotype. The group also identified a significantly higher expression of SCUBE1 and D-dimer in breast-cancer patients, suggesting SCUBE1 is responsible for hypercoagulability [59]. Karagüzel et al. (2017) identified SCUBE1 as a promising biomarker in the diagnosis and prognosis of renal tumors. They identified a significant increase in the expression of SCUBE1 in renal = cell carcinoma compared with soluble urokinase plasminogen activator receptor and carbonic anhydrase IX in plasma samples [9]. Mentese et al. (2012) demonstrated SCUBE1 to be a sensitive and specific biomarker for gastric cancer having higher SCUBE1 levels compared to those of the controls [58]. Prognostic multigene expression profiles based on the levels of tumor gene expression estimated SCUBE2 along with 20 other signature genes as hallmarks of cancer-related variability in breast-cancer symptoms. This gene set was identified to affect pathways involved in cancer development and progression [64]. Skrzypczak et al. (2013) reported significantly lower expression of SCUBE2 in high-grade endometrial cancer (G3) in comparison to the postmenopausal endometrium or G1 tumors. The study found a significant positive correlation between SCUBE2 transcription and PR and ERα, and PTEN expression might serve as a potential prognostic or predictive marker in endometrial cancer [21]. Studies suggested SCUBE2 to be a prognostic and diagnostic biomarker in nasopharyngeal carcinoma and distant metastatic premenopausal patients [65,66]. SCUBE3 is also an independent poor prognostic factor in breast cancer [42]. Joosten et al. (2017) reviewed SCUBE3 as a promising prognostic promoter methylation marker for renal-cell carcinoma [67].

## 5. Role of SCUBE in Patient Survival

In a clinical study, SCUBE2 was suggested to have increased disease-free survival with poor overall survival in colorectal cancer [23]. Another study reported SCUBE2 association with reduced survival in breast cancer [68]. Kaplan–Meier data and univariate analysis demonstrated that colon-cancer patients with SCUBE2-positive tumors had better overall survival and disease-free survival in comparison to patients with SCUBE2-negative tumors. In gastric cancer, lower SCUBE2 expression exhibited significantly poor relapse-free survival and overall survival in patients [50]. Ottley et al. (2020) demonstrated the association of luminal marker SCUBE2 and FOXA1 with disease-specific survival (DFS) in bladder cancer [51]. Lower transcriptional and protein expression due to the hypermethylation of SCUBE2 has been reported in hepatocellular carcinoma patients. The lower methylation of SCUBE2-cg19000089 was significantly associated with better overall survival in HCC patients [69]. NSCLC patients with high SCUBE3 expression had significantly less survival time in comparison to that of patients with low SCUBE3 expression [54]. A study reported that the co-expression of Bcl2 and SCUBE2 is a notable factor for poor survival in estrogen-receptor-positive breast-cancer patients [70]. Breast-cancer patients expressing SCUBE2 have better DFS but not OS in comparison to SCUBE2-negative patients. However, multivariate analysis showed that the expression of the SCUBE2 protein is an independent prognostic marker for disease-free survival [71].

## 6. Conclusions and Future Directions

The altered expression of SCUBE family proteins and its association with cancer was demonstrated in a cellular contextually dependent manner. Most studies focus on the expression and function of SCUBE family in cancer. However, the mechanistic role of the SCUBE family in other tumors needs further exploration. Conditional engineered mouse models could provide valuable information on the function of SCUBE and the mechanisms by which SCUBE contributes to cancer progression. Systematic approaches are needed to screen the substrates and interactions of SCUBE family proteins with other proteins that could provide information on their physiological role and mapping in various cancers. Studies on the SCUBE family showed potential towards their development as a diagnostic or prognostic biomarker in cancer. Clinical studies with larger samples are needed on SCUBE and their association with disease risk, overall survival, and therapeutic response in cancer patients. It is also important to determine the reason why the SCUBE family exhibits a dual role in certain types of cancer.

Reports on the small interfering RNA (siRNA)-mediated targeting of oncogenic function of SCUBE proteins in cancer indicate its druggable potential, but in-depth studies are required in this context. Targeting SCUBE family proteins with small molecules might be a promising strategy in anticancer drug discovery, at least in those cancers where the overexpression of these proteins is reported. This strategy is more useful in those cancers including NSCLC, glioma, and breast-cancer subtypes other than TNBC, colorectal cancer, and renal-cell carcinoma, where SCUBE family proteins play a role as tumor suppressors. Targeting downstream signaling molecules or SCUBE–other protein interactions by small molecules is also an interesting strategy in cancer drug discovery. Furthermore, the effect of small molecules on SCUBE-mediated cancer stemness, self-renewal, and chemotherapy resistance remains unexplored. The prevalence of SCUBE deregulation during tumorigenesis and cancer progression demands their further investigation and the development of rationally designed therapeutic strategies.

## Figures and Tables

**Table 1:** SCUBE family disease association.

SCUBE	Disease Class	Semantic Type	Association Type	References
SCUBE1				
RCC	Neoplasms, female urogenital diseases, pregnancy complications, male urogenital diseases.	Neoplastic process	Biomarker	[9]
Diabetic retinopathy	Eye diseases, endocrine system diseases, cardiovascular diseases.	Disease or syndrome	Altered expression	[10]
SLE	Skin and connective tissue diseases, immune system diseases.	Disease or syndrome	Genetic variation	[11]
Degenerative polyarthritis	Musculoskeletal diseases.	Disease or syndrome	Genetic variation	[12]
Psoriasis	Skin and connective tissue diseases.	Disease or syndrome	Altered expression	[13]
Thrombosis	Cardiovascular diseases.	Pathologic function	Altered expression	[5]
Gestational diabetes	Nutritional and metabolic diseases, female urogenital diseases and pregnancy complications, endocrine system diseases.	Disease or syndrome	Altered expression	[14]
ST segment elevation myocardial infarction	Pathological conditions, signs and symptoms, cardiovascular diseases.	Disease or syndrome	Altered expression	[15]
Venous thromboembolism	Cardiovascular diseases.	Disease or syndrome	Genetic variation	[16]
Molar incisor hypo-mineralization	Congenital, hereditary, and neonatal diseases and abnormalities, stomatognathic diseases.	Disease or syndrome	Genetic variation	[17]
SCUBE2				
Mammary neoplasms	Neoplasms, skin and connective tissue diseases.	Neoplastic process	Biomarker	[18]
Triple negative breast neoplasms	Neoplasms, skin and connective tissue diseases.	Neoplastic process	Altered expression	[19]
Tumor angiogenesis	Pathological conditions, signs, and symptoms.	Neoplastic process	Biomarker	[7]
Squamous cell carcinoma of mouth	Digestive system diseases, neoplasms, stomatognathic diseases.	Neoplastic process	Altered expression	[20]
Malignant neoplasm of endometrium	Neoplasms, female urogenital diseases and pregnancy complications.	Neoplastic process	Biomarker	[21]
NSCLC	Neoplasms, respiratory tract diseases.	Neoplastic process	Altered expression	[22]
CRC	Digestive system diseases, neoplasms.	Neoplastic process	Biomarker	[23]
Glioma	Neoplasms.	Neoplastic process	Biomarker	[24]
Thrombosis	Cardiovascular diseases.	Pathologic function	Altered expression	[5]
SCUBE3				
SACC	Pathological conditions, signs and symptoms, neoplasms.	Neoplastic process	Biomarker	[25]
Osteosarcoma of bone	Neoplasms.	Neoplastic process	Altered expression	[26]
RCC	Neoplasms, female urogenital diseases and pregnancy complications, male urogenital diseases.	Neoplastic process	Genetic variation	[9,27]

Note: data collected from DisGeNET database. RCC, renal cell carcinoma; NSCLC, nonsmall-cell lung carcinoma; CRC, colorectal carcinoma; SACC, salivary adenoid cystic carcinoma; SLE, systemic lupus erythematous.

**Table 2:** Association of SCUBE (1, 2, or 3) with other proteins and putative outcomes.

SCUBE Family Protein	Associated Protein and Its Function	Study on Association between SCUBE and Proteins	Mechanism	References
SCUBE1	SCUBE3	Inverse relation between SCUBE1 and SCUBE3 protein in synovial angiogenesis driven RA	SCUBE1 antagonizes BMP2 signaling, suppressing BMP2-induced phospho-Smad1/5/8 expression level, whereas Scube3 functions as a ligand for endogenous TGFβ receptor that increases Smad2/3 phosphorylation, and thus upexpresses target genes involved in angiogenesis.	[28]
SCUBE1	ITM2A is the BRICHOS (consisting of variety of proteins, each of 100 amino acids) resembling proteins, and structurally related to ITM2B and ITM2C. It is generally expressed in T-lineage among hematopoietic cells and is induced by MHC-mediated positive selection. It is involved in osteo- and chondrogenic differentiation. The downregulation of this gene causes ankylosing spondylitis, grave disease pathogenesis, and ovarian cancer.	SCUBE1 and ITM2A both downregulated, related to the proliferation of porcine follicular granulosa cells in an in vitro model.		[29,30]
SCUBE1	C1s is a single-chain glycoprotein and a serine protease that performs the catalytic function of the C1 complex.	C1s is a part of SCUBE1 that helps in executing the functions of SCUBE1.		[31]
SCUBE1	C1r is the serine protease enzyme that activates the proteolytic activity of complement complex C1.	C1r is a part of SCUBE1 that helps in executing the functions of SCUBE1.		[32]
SCUBE2	IHH is a member of the hedgehog signaling family that regulates embryonic morphogenesis and bone development.	SCUBE 2 is the most potent modulator of IIH signaling.	SCUBE 2 exerts an osteogenic function by increasing the IHH-mediated osteoblast differentiation in an in vitro mouse model. The deregulation of SCUBE2 impedes IHH signaling-mediated chondrocyte differentiation and proliferation, and thus bone formation.	[33]
SCUBE2	SHH is the secreted protein plays an essential role during embryo development, and aberrant expression leads to the generation of diseases, including atherosclerosis and cancer.	Overexpression of SCUBE2 inhibited the expression of Shh signaling pathway, thereby inhibiting proliferation, migration, and invasion in glioma and NSCLC cells.	Overexpression of SCUBE2 decreases the expression of Smo, Gli1, and Ptch1 (components of Shh signaling pathway), which ultimately decreases Shh signaling expression in glioma and NSCLC.	[22,24]
SCUBE2	GRB7 is an adapter protein that interacts with various receptor tyrosine kinases and modulates downstream signaling molecules such as AKT1, MAPK, and STAT3. This protein plays an important role in cell migration by binding with FAK.	SCUBE2 and GRB7 are both signature genes involved in the poor survival of breast-cancer patients in CNV-based assay of oncotype DX genes.	-	[34]
SCUBE2	CTSV is a lysosomal cysteine protease particularly found in human testes, thymus, and corneal epithelium. Overexpression of CTSV causes colorectal and breast cancers, and squamous cell carcinoma.	SCUBE2 and CTSV are also both signature genes involved in the poor survival of breast-cancer patients in CNV-based assay of Oncotye DX genes	-	[34]
SCUBE3	MMP2/9 is an enzyme that breaks ECM during normal developmental processes (embryo development, reproduction, etc.). In cancer, it allows for the primary tumor cells to migrate to form metastatic cancer cells. TGFBR2 is a transmembrane ser/thr kinase receptor expressed in all cell types such as fibroblasts. It is a tumor-suppressor gene. Loss of TGFBR2 expression is associated with many pathological conditions, including cancer.	SCUBE3, MMP2/9, and TGFBR2 are associated with the invasiveness of lung cancer.	The secreted form of SCUBE3 cleaved by MMP2/9 into N-and C-terminal. C fragments of the SCUBE3 bound to TGFBR2 activate TGF-β signaling and increase the transcription of Smad2/3, and upregulate the expression of genes involved in lung-cancer cell invasion.	[35]

Note: Interaction of SCUBE family with other proteins was experimentally studied. ITM2A, integral membrane protein 2A; BMP2, bone morphogenetic protein 2; C1s, complement component 1s; C1r-complement component 1r; IHH, Indian hedgehog; CTSV, cathepsin V; MMP2/9, matrix-metalloproteinase 2/9; TGFBR2, transforming growth factor-beta receptor 2; ECM, extracellular matrix; CNV, copy number variants; GRB7, growth factor bound protein 7; Akt, protein kinase; MAPK, mitogen activated protein kinase; STAT3, signal transducer and activator of transcription 3; FAK, focal adhesion kinase; NSCLC, nonsmall-cell lung cancer; RA, rheumatoid arthritis; MHC, major histocompatibility complex; SHH, sonic hedgehog.

**Table 3:** Role of SCUBE1/2/3 in cancer.

Cancer	SEL	Effect	Targets	Pathway	References
SCUBE2					
NSCLC	D	Promotes proliferation, migration, and invasion.	Ptch1, Shh, Smo, Gli1	Hedgehog	[22]
Glioma	D	Promotes proliferation, migration, and invasion.	Ptch, Shh, Smo, Gli1	Hedgehog	[24]
BCa	D	Promotes proliferation, migration, and invasion, and induces apoptosis.	MiR-155-5p, BMP, TGF-β, FOXA1	MiR-155-5p/SCUBE2 axis, β-catenin	[18,36]
BCaSC	U	Enhances stemness phenotype, self-renewal, aggressiveness, and EMT.	Sox2, Oct4, Nanog, Vimentin	Notch	[19]
TA	U	Increases tube formation, proliferation, and migration.	VEGF, VEGFR2	PI3K/Akt, MAPK	[44]
CRC	D	Promotes proliferation, migration, invasion, and apoptosis inhibition.	-	-	[23]
GC	D	Increases lymph-node metastasis, high-grade tumors, T3 or T4 lesions, distant metastasis, and vascular invasion	-	-	[50]
SCUBE1					
PCOS	U	-	TNF-α and IL-1β	-	[57]
RCC	U	-	-	-	[9]
GC	U	-	-	-	[58]
BC	U	-	-	-	[59]
SCUBE3					
LC	U	Increases metastasis.	MMP2/9, TGF-β, Cadherin, Vimentin	EMT	[35]
BlCa	U	Increases proliferation and metastasis.	E-Cadherin, MMP2/9	-	[42]
SACC	U	Increases SACC proliferation.	miR-885-5p	-	[25]

TA, tumor angiogenesis; NSCLC, nonsmall-cell lung cancer; BCa, breast cancer; BCaSC, breast-cancer stem cell; GC, gastric cancer; CRC, colorectal carcinoma; Ptch1, patched homolog 1; Shh, sonic hedgehog; Smo, Smoothened; Gli1, Gli family zinc finger 1; BMP, bone morphogenetic protein; TGF-β, tumor growth factor-β; FOXA1, forkhead box protein A1; VEGF, vascular endothelial growth factor; VEGFR, vascular endothelial growth factor receptor 2; PI3K/Akt, phosphotidylinositol 3-kinase/protein kinase B; MAPK, mitogen activated protein kinase; SEL, SCUBE expression level; LC, lung cancer; TNF-α, tumor necrosis factor-α; IL-1β, interleukin-1β; BLC, bladder cancer; SACC, salivary adenoid cystic carcinoma; MMP2/9, matrix-metalloproteinase 2/9; EMT, epithelial–mesenchymal transition; RCC, renal-cell cancer; U, up; D, down.

## Data Availability

Not applicable.

## Abbreviations

SCUBE	Signal peptide CUB and EGF-like domain-containing protein
BMP	Bone morphogenetic protein
NSCLC	Nonsmall-cell lung cancer
VEGF	Vascular endothelial growth factor
FOXO1	Forkhead box protein 1
TGF-β	Transforming growth factor-beta
BC	Breast cancer
TNBC	Triple-negative breast cancer
lncRNA	Long noncoding RNA

## References

[1] Tsai M.-T., Cheng C.-J., Lin Y.-C., Chen C.-C., Wu A.-R., Wu M.-T., Hsu C.-C., Yang R.-B. (2009). Isolation and characterization of a secreted, cell-surface glycoprotein SCUBE2 from humans. Biochem. J..

[2] Grimmond S., Larder R., Van Hateren N., Siggers P., Hulsebos T.J., Arkell R., Greenfield A. (2000). Cloning, Mapping, and Expression Analysis of a Gene Encoding a Novel Mammalian EGF-Related Protein (SCUBE1). Genomics.

[3] Grimmond S., Larder R., Van Hateren N., Siggers P., Morse S., Hacker T., Arkell R., Greenfield A. (2001). Expression of a novel mammalian epidermal growth factor-related gene during mouse neural development. Mech. Dev..

[4] Tu C.-F., Su Y.-H., Huang Y.-N., Tsai M.-T., Li L.-T., Chen Y.-L., Cheng C.-J., Dai D.-F., Yang R.-B. (2006). Localization and characterization of a novel secreted protein SCUBE1 in human platelets. Cardiovasc. Res..

[5] Yang R.-B., Ng C.K.D., Wasserman S.M., Colman S.D., Shenoy S., Mehraban F., Kömüves L.G., Tomlinson J.E., Topper J.N. (2002). Identification of a Novel Family of Cell-surface Proteins Expressed in Human Vascular Endothelium. J. Biol. Chem..

[6] Jakobs P., Exner S., Schürmann S., Pickhinke U., Bandari S., Ortmann C., Kupich S., Schulz P., Hansen U., Seidler D.G. (2014). Scube2 enhances proteolytic Shh processing from the surface of Shh-producing cells. J. Cell Sci..

[7] Lin Y.-C., Chao T.-Y., Yeh C.-T., Roffler S.R., Kannagi R., Yang R.-B. (2017). Endothelial SCUBE2 Interacts with VEGFR2 and Regulates VEGF-Induced Angiogenesis. Arter. Thromb. Vasc. Biol..

[8] Wu B.-T., Su Y.-H., Tsai M.-T., Wasserman S.M., Topper J.N., Yang R.-B. (2004). A Novel Secreted, Cell-surface Glycoprotein Containing Multiple Epidermal Growth Factor-like Repeats and One CUB Domain Is Highly Expressed in Primary Osteoblasts and Bones. J. Biol. Chem..

[9] Karagüzel E., Menteşe A., Kazaz I.O., Demir S., Örem A., Okatan A.E., Altay D.U., Yaman S. (2017). SCUBE1: A promising biomarker in renal cell cancer. Int. Braz J. Urol.

[10] Icel E., Icel A., Mertoglu C., Tasli N.G., Karakurt Y., Ucak T., Gunay M. (2019). Serum SCUBE-1 levels in patients with diabetic retinopathy. Int. Ophthalmol..

[11] Harley J.B., Alarcón-Riquelme M.E., Criswell L.A., Jacob C.O., Kimberly R.P., Moser K.L., Tsao B.P., Vyse T.J., Langefeld C.D., International Consortium for Systemic Lupus Erythematosus Genetics (SLEGEN) (2008). Genome-wide association scan in women with systemic lupus erythematosus identifies susceptibility variants in ITGAM, PXK, KIAA1542 and other loci. Nat. Genet..

[12] Tachmazidou I., Hatzikotoulas K., Southam L., Esparza-Gordillo J., Haberland V., Zheng J., Johnson T., Koprulu M., Zengini E., Steinberg J. (2019). Identification of new therapeutic targets for osteoarthritis through genome-wide analyses of UK Biobank data. Nat. Genet..

[13] Capkin A.A., Demir S., Mentese A., Bulut Ç., Ayar A. (2017). Can signal peptide-CUB-EGF domain-containing protein (SCUBE) levels be a marker of angiogenesis in patients with psoriasis?. Arch. Dermatol. Res..

[14] Tekin Y.B., Erin K.B., Yilmaz A. (2019). Evaluation of SCUBE-1 levels as a placental dysfunction marker at gestational diabetes mellitus. Gynecol. Endocrinol..

[15] Bolayır H.A. (2017). The role of SCUBE1 in the pathogenesis of no-reflow phenomenon presenting with ST segment elevation myocardial infarction. Anatol. J. Cardiol..

[16] Heit J.A., Cunningham J.M., Petterson T.M., Armasu S.M., Rider D.N., DE Andrade M. (2011). Genetic variation within the anticoagulant, procoagulant, fibrinolytic and innate immunity pathways as risk factors for venous thromboembolism. J. Thromb. Haemost..

[17] Kühnisch J., Thiering E., Heitmüller D., Tiesler C.M.T., Grallert H., Heinrich-Weltzien R., Hickel R., Heinrich J., The GINI-10 plus study group, The LISA-10 plus study group (2013). Genome-wide association study (GWAS) for molar–incisor hypomineralization (MIH). Clin. Oral Investig..

[18] Lin Y.-C., Chen C.-C., Cheng C.-J., Yang R.-B. (2011). Domain and Functional Analysis of a Novel Breast Tumor Suppressor Protein, SCUBE2. J. Biol. Chem..

[19] Chen J.-H., Kuo K.-T., Bamodu O.A., Lin Y.-C., Yang R.-B., Yeh C.-T., Chao T.-Y. (2018). Upregulated SCUBE2 expression in breast cancer stem cells enhances triple negative breast cancer aggression through modulation of notch signaling and epithelial-to-mesenchymal transition. Exp. Cell Res..

[20] Parris T.Z., Aziz L., Kovács A., Hajizadeh S., Nemes S., Semaan M., Chen C.Y., Karlsson P., Helou K. (2014). Clinical relevance of breast cancer-related genes as potential biomarkers for oral squamous cell carcinoma. BMC Cancer.

[21] Skrzypczak M., Lattrich C., Häring J., Schüler S., Ortmann O., Treeck O. (2013). Expression of SCUBE2 gene declines in high grade endometrial cancer and associates with expression of steroid hormone receptors and tumor suppressor PTEN. Gynecol. Endocrinol..

[22] Yang B., Miao S., Li Y. (2018). SCUBE2 inhibits the proliferation, migration and invasion of human non-small cell lung cancer cells through regulation of the sonic hedgehog signaling pathway. Gene.

[23] Song Q., Li C., Feng X., Yu A., Tang H., Peng Z., Wang X. (2015). Decreased expression of SCUBE2 is associated with progression and prognosis in colorectal cancer. Oncol. Rep..

[24] Guo E., Liu H., Liu X. (2017). Overexpression of SCUBE2 Inhibits Proliferation, Migration, and Invasion in Glioma Cells. Oncol. Res. Featur. Preclin. Clin. Cancer Ther..

[25] Han N., Lu H., Zhang Z., Ruan M., Yang W., Zhang C. (2018). Comprehensive and in-depth analysis of microRNA and mRNA expression profile in salivary adenoid cystic carcinoma. Gene.

[26] Liang W., Yang C., Peng J., Qian Y., Wang Z. (2015). The Expression of HSPD1, SCUBE3, CXCL14 and Its Relations with the Prognosis in Osteosarcoma. Cell Biophys..

[27] Morris M.R., Ricketts C.J., Gentle D., McRonald F., Carli N., Khalili H., Brown M., Kishida T., Yao M., Banks R.E. (2010). Genome-wide methylation analysis identifies epigenetically inactivated candidate tumour suppressor genes in renal cell carcinoma. Oncogene.

[28] Yang M., Guo M., Hu Y., Jiang Y. (2013). Scube regulates synovial angiogenesis-related signaling. Med. Hypotheses.

[29] Tai T.-S., Pai S.-Y., Ho I.-C. (2014). Itm2a, a Target Gene of GATA-3, Plays a Minimal Role in Regulating the Development and Function of T Cells. PLoS ONE.

[30] Kulus M., Sujka-Kordowska P., Konwerska A., Celichowski P., Kranc W., Kulus J., Piotrowska-Kempisty H., Antosik P., Bukowska D., Iżycki D. (2019). New Molecular Markers Involved in Regulation of Ovarian Granulosa Cell Morphogenesis, Development and Differentiation during Short-Term Primary In Vitro Culture—Transcriptomic and Histochemical Study Based on Ovaries and Individual Separated Follicles. Int. J. Mol. Sci..

[31] Gal P. (2002). C1s, the protease messenger of C1. Structure, function, and physiological significance. Immunobiology.

[32] Arlaud G.J., Rossi V., Thielens N., Gaboriaud C., Bersch B., Hernandez J.-F. (1998). Structural and Functional Studies on C1r and C1s: New Insights into the Mechanisms Involved in C1 Activity and Assembly. Immunobiology.

[33] Lin Y.-C., Roffler S.R., Yan Y.-T., Yang R.-B. (2015). Disruption of *Scube2* Impairs Endochondral Bone Formation. J. Bone Miner. Res..

[34] Ahmed W., Malik M.F.A., Saeed M., Haq F. (2018). Copy number profiling of Oncotype DX genes reveals association with survival of breast cancer patients. Mol. Biol. Rep..

[35] Wu Y.-Y., Peck K., Chang Y.-L., Pan S.-H., Cheng Y.-F., Lin J.-C., Yang R.-B., Hong T.-M., Yang P.-C. (2011). SCUBE3 is an endogenous TGF-β receptor ligand and regulates the epithelial-mesenchymal transition in lung cancer. Oncogene.

[36] Cheng C.-J., Lin Y.-C., Tsai M.-T., Chen C.-S., Hsieh M.-C., Chen C.-L., Yang R.-B. (2009). SCUBE2 Suppresses Breast Tumor Cell Proliferation and Confers a Favorable Prognosis in Invasive Breast Cancer. Cancer Res..

[37] Andres S.A., Wittliff J.L. (2012). Co-expression of genes with estrogen receptor-α and progesterone receptor in human breast carcinoma tissue. Horm. Mol. Biol. Clin. Investig..

[38] Fei H., Chen S., Xu C. (2020). RNA-sequencing and microarray data mining revealing: The aberrantly expressed mRNAs were related with a poor outcome in the triple negative breast cancer patients. Ann. Transl. Med..

[39] Jinesh G.G., Flores E.R., Brohl A.S. (2018). Chromosome 19 miRNA cluster and CEBPB expression specifically mark and potentially drive triple negative breast cancers. PLoS ONE.

[40] Kadio B., Yaya S., Basak A., Djè K., Gomes J., Mesenge C. (2016). Calcium role in human carcinogenesis: A comprehensive analysis and critical review of literature. Cancer Metastasis Rev..

[41] Heng L., Jia Z., Sun J., Zhao Y., Zhang K., Zhu Y., Lu S. (2020). Integrated Analysis of Competing Endogenous RNAs Network Reveals Potential Signatures in Osteosarcoma Development. Technol. Cancer Res. Treat..

[42] Huo Q., He X., Li Z., Yang F., He S., Shao L., Hu Y., Chen S., Xie N. (2021). SCUBE3 serves as an independent poor prognostic factor in breast cancer. Cancer Cell Int..

[43] Orr B., Grace O.C., Brown P., Riddick A.C.P., Stewart G.D., Franco O.E., Hayward S., Thomson A.A. (2012). Reduction of pro-tumorigenic activity of human prostate cancer-associated fibroblasts using Dlk1 or SCUBE1. Dis. Model. Mech..

[44] Lin Y.-C., Liu C.-Y., Kannagi R., Yang R.-B. (2018). Inhibition of Endothelial SCUBE2 (Signal Peptide-CUB-EGF Domain-Containing Protein 2), a Novel VEGFR2 (Vascular Endothelial Growth Factor Receptor 2) Coreceptor, Suppresses Tumor Angiogenesis. Arter. Thromb. Vasc. Biol..

[45] Jiang W., Han X., Wang J., Wang L., Xu Z., Wei Q., Zhang W., Wang H. (2019). *miR-22* enhances the radiosensitivity of small-cell lung cancer by targeting the *WRNIP1*. J. Cell. Biochem..

[46] Kim C., Paik S. (2010). Gene-expression-based prognostic assays for breast cancer. Nat. Rev. Clin. Oncol..

[47] Sparano J.A., Paik S. (2008). Development of the 21-Gene Assay and Its Application in Clinical Practice and Clinical Trials. J. Clin. Oncol..

[48] Lin Y.-C., Lee Y.-C., Li L.-H., Cheng C.-J., Yang R.-B. (2013). Tumor suppressor *SCUBE2* inhibits breast-cancer cell migration and invasion through the reversal of epithelial-mesenchymal transition. J. Cell Sci..

[49] Shen Y., Zhang M., Da L., Huang W., Zhang C. (2020). Circular RNA circ_SETD2 represses breast cancer progression via modulating the miR-155-5p/SCUBE2 axis. Open Med..

[50] Wang X., Zhong R.-Y., Xiang X.-J. (2018). Reduced expression of SCUBE2 predicts poor prognosis in gastric cancer patients. Int. J. Clin. Exp. Pathol..

[51] Islam S., Mokhtari R., Noman A., Uddin M., Rahman M., Azadi M., Zlotta A., van der Kwast T., Yeger H., Farhat W. (2015). Sonic hedgehog (Shh) signaling promotes tumorigenicity and stemness via activation of epithelial-to-mesenchymal transition (EMT) in bladder cancer. Mol. Carcinog..

[52] Sheng J., Shi W., Guo H., Long W., Wang Y., Qi J., Liu J., Xu Y. (2019). The Inhibitory Effect of (−)-Epigallocatechin-3-Gallate on Breast Cancer Progression via Reducing SCUBE2 Methylation and DNMT Activity. Molecules.

[53] Chou C.-H., Cheng Y.-F., Siow T.Y., Kumar A., Peck K., Chang C. (2013). SCUBE3 regulation of early lung cancer angiogenesis and metastatic progression. Clin. Exp. Metastasis.

[54] Zhao C., Qin Q., Wang Q., Zhang J., Xu Y., Li W., Gu M., Chen S., Deng A. (2013). SCUBE3 overexpression predicts poor prognosis in non-small cell lung cancer. Biosci. Trends.

[55] Kallarackal J., Burger F., Bianco S., Romualdi A., Schad M. (2020). A 3-gene biomarker signature to predict response to taxane-based neoadjuvant chemotherapy in breast cancer. PLoS ONE.

[56] Shuai C., Yuan F., Liu Y., Wang C., Wang J., He H. (2021). Estrogen receptor—positive breast cancer survival prediction and analysis of resistance–related genes introduction. PeerJ.

[57] Erol O., Ellidağ H.Y., Özel M.K., Derbent A.U., Eren E., Yılmaz N. (2018). Circulating SCUBE1 levels in women with polycystic ovary syndrome. J. Turk. Soc. Obstet. Gynecol..

[58] Mentese A., Fidan E., Sumer A.U., Karahan S.C., Sonmez M., Altay D.U., Kavgaci H., Alver A. (2012). Is SCUBE 1 a new biomarker for gastric cancer?. Cancer Biomark..

[59] Topcu T.O., Kavgaci H., Ozdemir F., Aksoy A., Erdem D., Mentese A., Yaman H., Tufan G., Orem A., Aydin F. (2015). Elevated Serum Levels of SCUBE1, a Marker for Coagulation, in Patients with Breast Cancer. Tohoku J. Exp. Med..

[60] Diamanti-Kandarakis E., Paterakis T., Kandarakis H.A. (2006). Indices of Low-Grade Inflammation in Polycystic Ovary Syndrome. Ann. N. Y. Acad. Sci..

[61] Akdoğan E., Ayaz T., Kırbaş A., Rakıcı H. (2019). Is SCUBE1 helpful to predict the arterial thrombotic risk in patients with multi-ple myeloma: A preliminary study. Hippokratia.

[62] Rickles F.R., Patierno S., Fernandez P.M. (2003). Tissue Factor, Thrombin, and Cancer. Chest.

[63] Dai D.-F., Thajeb P., Tu C.-F., Chiang F.-T., Chen C.-H., Yang R.-B., Chen J.-J. (2008). Plasma Concentration of SCUBE1, a Novel Platelet Protein, Is Elevated in Patients with Acute Coronary Syndrome and Ischemic Stroke. J. Am. Coll. Cardiol..

[64] Koleck T.A., Conley Y.P. (2016). Identification and prioritization of candidate genes for symptom variability in breast cancer survivors based on disease characteristics at the cellular level. Breast Cancer Targets Ther..

[65] Chen Y., Zhou C., Li H., Li H., Li Y. (2021). Identifying Key Genes for Nasopharyngeal Carcinoma by Prioritized Consensus Differentially Expressed Genes Caused by Aberrant Methylation. J. Cancer.

[66] Ni H., Kumbrink J., Mayr D., Seiler A., Hagemann F., Degenhardt T., Sagebiel S., Würstlein R., Kates R., Harbeck N. (2021). Molecular Prognostic Factors for Distant Metastases in Premenopausal Patients with HR+/HER2− Early Breast Cancer. J. Pers. Med..

[67] Joosten S.C., Deckers I.A., Aarts M.J., Hoeben A., Van Roermund J.G., Smits K.M., Melotte V., Van Engeland M., Tjan-Heijnen V.C. (2017). Prognostic DNA methylation markers for renal cell carcinoma: A systematic review. Epigenomics.

[68] Fatima A., Tariq F., Malik M.F.A., Qasim M., Haq F. (2017). Copy Number Profiling of MammaPrint™ Genes Reveals Association with the Prognosis of Breast Cancer Patients. J. Breast Cancer.

[69] Zhen L., Ning G., Wu L., Zheng Y., Yang F., Chen T., Xu W., Liu Y., Xie C., Peng L. (2020). Prognostic value of aberrantly expressed methylation genes in human hepatocellular carcinoma. Biosci. Rep..

[70] Esmaeili R., Mohammadi S., Jafarbeik-Iravani N., Yadegari F., Olfatbakhsh A., Mazaheri M., Kaviani A., Rezaee M., Majidzadeh-A K. (2021). Expression of SCUBE2 and BCL2 Predicts Favorable Response in ERα Positive Breast Cancer. Arch. Iran. Med..

[71] Poorhosseini S.M., Hashemi M., Olyaei N.A., Izadi A., Moslemi E., Ravesh Z., Hashemi-Gorji F., Kheiri H.R., Yassaee V.R. (2016). New Gene Profiling in Determination of Breast Cancer Recurrence and Prognosis in Iranian Women. Asian Pac. J. Cancer Prev..

